# Durability and effectiveness of dual vs. triple therapy and tablet simplification in ART: findings from the Italian MOSAICO study

**DOI:** 10.3389/fphar.2025.1633968

**Published:** 2025-08-06

**Authors:** Daniele Mengato, Giacomo Berti, Andrea Francavilla, Silvia Michielan, Linda Cappellazzo, Laura Agnoletto, Maria Chiara Silvani, Marco Chiumente, Dario Gregori, Maria Mazzitelli, Francesca Venturini, Anna Maria Cattelan, the SIFaCT MOSAICO Study Group, Salvatore Agosta

**Affiliations:** ^1^ Hospital Pharmacy Unit, Azienda Ospedale-Università Padova, Padova, Italy; ^2^ Unit of Biostatistics, Epidemiology and Public Health, Department of Cardiac Thoracic Vascular Sciences and Public Health, University of Padova, Padova, Italy; ^3^ Pharmaceutical Department, Azienda AUSL of Modena, Modena, Italy; ^4^ Hospital Pharmacy, Hospital of Bolzano (SABES-ASDAA), Bolzano, Italy; ^5^ Hospital Pharmacy Unit, Azienda Ulss 5 Polesana, Rovigo, Italy; ^6^ Hospital Pharmacy Unit, Azienda USL Romagna, Ravenna, Italy; ^7^ Società Italiana di Farmacia Clinica e Terapia (SIFaCT), Turin, Italy; ^8^ Infectious and Tropical Diseases Unit, Azienda Ospedale-Università Padova, Padova, Italy

**Keywords:** people with HIV, single tablet regimen, dual therapy, HIV treatment, switch therapy, optimization

## Abstract

**Introduction:**

Treatment optimization in people with HIV (PWH) has increasingly focused on reducing drug burden and improving regimen simplicity. However, comparative real-world evidence on dual therapy (DT) vs. triple therapy (TT), and single-tablet regimens (STR) vs. multi-tablet regimens (MTR), remains limited.

**Methods:**

The MOSAICO study is a multicenter, retrospective observational analysis conducted across 20 centers, including people with HIV on a stable virological suppression who switched antiretroviral therapy between 2017 and 2019. People were followed-up up to 48 months post-switch. Comparative analyses assessed virological suppression (HIV-RNA <50 copies/mL), CD4^+^ T cell count, CD4/CD8 ratio, and treatment discontinuation. Propensity score weighting was applied to adjust for baseline differences.

**Results:**

Four hundred ninety-one PWH were included. Both DT and triple therapy groups maintained high levels of virological suppression over 48 months (12 months: 97.1% vs. 91.6%; 24 months: 100% vs. 95.6%; 36 months: 100% vs. 96.9%; 48 months: 100% vs. 100%). From 24 months onward, all persons living with HIV remaining on their respective regimens achieved full virological suppression. Immunological recovery (CD4^+^ count and CD4/CD8 ratio) was comparable across groups, although TT and MTR groups showed greater increases from lower baselines. STRs demonstrated significantly greater treatment durability than MTRs (aHR = 0.56, 95% CI: 0.32–0.97; p = 0.039), while no significant difference in persistence was found between DT and TT. INSTI-based regimens were predominant in DT and MTR arms (DT vs. TT: 84% vs. 46.52%, p < 0.01; MTR vs. STR: 59.38% vs. 47.14%, p < 0.01).

**Discussion:**

The real-world effectiveness of both dual and triple therapies when tailored to appropriate person profiles. STRs offer enhanced long-term persistence compared to MTRs, supporting treatment simplification strategies. These results reinforce the importance of individualized treatment approaches balancing clinical effectiveness with person-centered considerations such as pill burden and tolerability. Limitations include the retrospective design and the lack of quality-of-life data, which may affect interpretation of patient-centered outcomes. Future efforts should expand access to dual-agent STR to further improve Antiretroviral Therapy outcomes.

## 1 Introduction

### 1.1 Advances antiretroviral therapy (ART) and need for optimization

Thanks to more effective and tolerable combination ART, HIV management significantly evolved over the past decades, becoming a manageable chronic condition (Iacob et al., 2017; Saag et al., 2020), and with a remarkable increase of life expectancy. However, as life expectancy increased, the burden of comorbidities—especially cardiovascular, metabolic, and oncological—also grew. This is likely due to persistent inflammation and immune activation, even in the context of virological suppression (Montes et al., 2025; Nganou-Makamdop, 2025). For this reason, multidimensional clinic have been implemented in order to promote screening for the main comorbidities, reduce drug-related issues, such as interactions and side effect, and to provide a better quality of care (Mazzitelli et al., 2024; Pereira et al., 2022).

### 1.2 Dual versus triple therapy: efficacy and long-term considerations

Traditionally recognized and recommended ART typically involve a combination of antiretroviral agents that work synergistically to suppress viral replication, thereby preventing disease progression and reducing transmission risk (NIH, 2024). Although the reasons for treatment optimization may vary, two key aspects are central to ART regimen design: the number of drugs—dual therapy (DT) *versus* triple therapy (TT)—and their formulation—single-tablet regimens (STR) *versus* multi-tablet regimens (MTR) (Cahn et al., 2019).

Triple therapy, consisting of three antiretroviral agents, had for a long time been the cornerstone of HIV treatment due to its robust efficacy in achieving viral suppression. However, concerns about long-term drug toxicity, adherence challenges, and cost have spurred interest in dual therapy regimens that utilize two antiretroviral agents (Simoni et al., 2019). Recent clinical trials have demonstrated the non-inferiority of certain dual therapy combinations in people with HIV (PWH) with a stable virological suppression (Jaeger et al., 2021; Llibre et al., 2023; van Wyk et al., 2020a; 2020b). A systematic review and meta-analysis encompassing 14 studies with 5,205 PWH found no significant difference in treatment failure rates between DT and TT at 48 weeks, but noted a higher rate of resistance-associated mutations in individuals on DT at 96 weeks, suggesting potential long-term considerations when opting for DT (Russo et al., 2024). Moreover, also in real-life and different cohorts this results on dual therapy was largely confirmed (Fabbiani et al., 2021; Mazzitelli et al., 2023).

### 1.3 Reducing pill burden in ART: single-tablet regimens (STRs) versus multi-tablet regimens (MTRs)

The complexity of ART regimens can significantly impact adherence, which is crucial for maintaining sustained viral suppression and preventing resistance. STRs, which combine multiple agents into one daily pill, were developed to simplify therapy and to optimize adherence. Several studies and meta-analyses have shown that STRs are associated with better adherence and virological outcomes compared to MTRs (Clay et al., 2018; Kapadia et al., 2018). Beyond clinical efficacy, STRs have also been linked to improved treatment satisfaction, reduced perceived burden, and lower healthcare costs and resource use (Aldir et al., 2014; Li et al., 2023), further supporting their role in treatment optimization.

### 1.4 Evidence gaps in real-world practice and study rationale

While randomized controlled trials (RCTs) provide critical evidence for regulatory approval and treatment guidelines, real-world studies offer complementary insights that are essential for optimizing clinical practice. Real-world data capture outcomes across heterogeneous patient populations often excluded from RCTs, including those with comorbidities, prior treatment experience, and diverse demographic characteristics (Hsu et al., 2023). A recent analysis of real-world evidence in HIV care highlighted that treatment outcomes in clinical practice may differ from those observed in highly controlled trial settings, particularly regarding adherence patterns, tolerability, and long-term persistence (Neesgaard et al., 2022). Additionally, real-world studies allow for extended follow-up periods that better reflect the lifelong nature of HIV management, providing valuable information about the durability of treatment effects beyond the typical 48–96 weeks endpoints of registration trials (Borghetti et al., 2019). The European AIDS Clinical Society has emphasized the importance of integrating evidence from both RCTs and real-world studies to develop comprehensive treatment guidelines that address the complex needs of diverse patient populations (Ryom et al., 2022).

Despite growing interest in treatment optimization, real-world data comparing dual *versus* triple therapies and single-*versus* multi-tablet regimens remain limited, particularly over long-term follow-up. RCTs have demonstrated non-inferiority of dual therapies and benefits of STRs, but their generalizability may be limited by strict inclusion criteria and short duration. In this context, we hypothesized that, in real-world settings, dual therapy and STRs would maintain comparable virological and immunological outcomes to standard regimens, with differences potentially emerging in treatment durability.

The MOSAICO study (Multicenter Observational Study on cArt Optimization in hiv patients) was designed to fill this gap by evaluating, at a national scale, the long-term real-world effectiveness and safety of DT vs. TT and STR vs. MTR in PWH with stable virological suppression, with the specific aim to assess clinical outcomes and treatment durability across a long-term, 48-month follow-up.

## 2 Materials and methods

### 2.1 Study design

This study is a multicenter, retrospective observational analysis aiming at comparing the effectiveness and safety of various cART in PWH who are on a stable virological suppression and have undergone therapeutic optimization. Data were collected from clinical records and linked across 20 Italian centers. Italy has a universal, publicly funded healthcare system that provides comprehensive HIV care—including ART access and monitoring—free of charge to all citizens and residents. HIV management is centralized in infectious disease units, often within tertiary hospitals, with a high degree of uniformity in clinical practice across regions.

Data were collected from clinical records of 20 infectious disease centers across Northern, Central, and Southern Italy, selected to ensure geographic diversity and representation of different healthcare settings. Participating centers were part of a national hospital pharmacist network coordinated by the Italian Society for Clinical Pharmacy and Therapeutic (Società Italiana di Farmacia Clinica e Terapia - SIFaCT).

The cohort reflects a broad cross-section of real-world clinical practice in Italy, including persons living with HIV from both urban and non-urban referral hospitals, making the sample representative of the national population of virologically suppressed PWH undergoing ART optimization during the study period.

Each participant was followed for up from first switch of optimization to at least 48 months following the optimization. The study data were managed using REDCap electronic data capture tools (Harris et al., 2009).

Inclusion criteria for participants were: age ≥18 years; initiation of cART between 1 January 2017 and 31 December 2019; a switch to therapy for the first time within the same period; virological suppression (HIV-RNA <50 copies/mL) at the time of the switch; and a minimum of 24 months of follow-up after the first switch (time zero). Exclusion criteria included: age <18 years and pregnancy or breastfeeding.

We included only persons living with HIV with at least 24 months of follow-up after ART switch to allow for meaningful evaluation of long-term virological, immunological, and persistence outcomes, which were central to the study objectives. This criterion ensured sufficient time to observe treatment durability and minimize short-term censoring effects.

The target population for this study includes adult people living with HIV (PWH) with sustained virological suppression who underwent treatment optimization for clinical, safety, or adherence-related reasons. While this follow-up requirement may limit generalizability to those with shorter treatment histories or more recent switches, it reflects a real-world population eligible for long-term therapeutic simplification and monitoring.

The primary objective of the study was to compare the effectiveness and safety of different cART optimization strategies. Specifically, two comparisons were made: PWH in treatment with a dual therapy (DT) *versus* PWH in treatment with a triple therapy (TT); and PWH in treatment with a single-table regimen (STR) *versus* PWH in treatment with a multi-tablet regimen (MTR).

### 2.2 Statistical methods

Patient characteristics at baseline, switch, and follow-up were summarized overall and by group. Group differences were assessed using Pearson’s Chi-squared or Fisher’s test for categorical variables and the Wilcoxon test for continuous variables.

To visualize cART regimen transitions, Sankey diagrams were generated with the *ggalluvial* R package, using data from each treatment switch to represent flows between regimens over time (Brunson and Read, 2023).

Treatment efficacy at 12-, 24-, 36-, and 48-month post-switch was evaluated *via* multivariable logistic regression, considering virological suppression (HIV-RNA <50 copies/mL), CD4^+^ ≥500 cells/mm^3^, and CD4/CD8 ratio ≥1. Censoring occurred due to death, loss to follow-up, or further regimen change.

Safety was assessed *via* treatment durability, defined as time to discontinuation due to clinical reasons (virological failure, toxicity, drug resistance, drug–drug interactions, or poor adherence), using Kaplan-Meier curves and Cox regression up to 24 months. Only reactive regimen changes between treatment categories in comparison were considered discontinuations - specifically, switches from DT to TT (or *vice versa*), or from MTR to STR, and *vice versa*. Censoring followed the same criteria as efficacy but excluded non-reactive regimen changes (e.g., optimization). The Integrase strand transfer inhibitors (INSTI) based regimen was the main covariate in both models. INSTI-based regimens were defined as antiretroviral treatment combinations containing an integrase strand transfer inhibitor (INSTI), including dolutegravir, bictegravir, or elvitegravir, in either single-tablet or multi-tablet formulations.

To adjust for confounding, propensity score weighting (Inverse Probability Weighting, IPW) with stabilized weights estimated *via* logistic regression was applied, including age ≥50, comorbidities, coinfections, Caucasian ethnicity, CD4^+^ ≥500 cells/mm^3^, and CD4/CD8 ≥1 at switch. Weights were trimmed at the 90th percentile to reduce outliers, and all models were weighted accordingly. Missing data were handled using listwise deletion. No imputation techniques were applied. This approach was chosen to ensure analytical consistency and interpretability of effect estimates. The number of excluded observations due to missing values is reported in the relevant tables.

Propensity score distributions before and after weighting are reported in Supplementary Figures S3A,B to evaluate balance and support the positivity assumption.

Analyses were conducted in R (v4.4.1) using the *survey*, *survival*, and *weightit* packages. A p-value <0.05 was considered statistically significant (Greifer, 2025; Lumley and Huang, 2024; R Core Team, 2021; Themeau and Grambsch, 2024).

## 3 Results

Over the study period, 419 people were included. At 36th months 52 persons living with HIV were lost to follow-up (12.4%), and at 48th months 110 additional persons living with HIV were lost to follow-up (38.66% overall lost in follow-up at 48th month) Cohort description overall and by treatment group is reported into Table 1. Most participants were male (79.71%), with a mean age of 42.63 years (Standard deviation (SD) 12.26) with 26.49% being over 50 years of age. Caucasians represented 80.91% of the population, followed by Afro-American/Afro (8.11%) and Latin/Hispanic (4.54%). At time of switch, 55.37% had at least one co-infection, with syphilis (17.66%) and Cytomegalovirus (CMV) (13.60%) being the most frequent. Hepatitis B virus (HBV) and Hepatitis C virus (HCV) co-infections were present in 6.92% and 8.11% of cases, respectively. Additionally, 53.22% of participants had at least one comorbidity, with cardiovascular disease (15.51%) being the most common one.

**TABLE 1:** Cohort features overall, and by treatment group.

		Comparison DT vs. TT			Comparison MTR vs. STR		
Characteristic	Overall N = 419^a^	DT N = 75^a^	TT N = 344^a^	p-value^b^	MTR N = 192^a^	STR N = 227^a^	p-value^b^
Gender				0.45			0.31
Female	80 (19.09%)	11 (14.67%)	69 (20.06%)		42 (21.88%)	38 (16.74%)	
Male	334 (79.71%)	64 (85.33%)	270 (78.49%)		147 (76.56%)	187 (82.38%)	
Transgender	5 (1.19%)	0 (0%)	5 (1.45%)		3 (1.56%)	2 (0.88%)	
Age				0.35			0.31
Median (Q1, Q3)	42.69 (34.20, 51.08)	42.91 (34.45, 53.30)	42.67 (34, 50.28)		42.75 (35.46, 51.40)	42.66 (32.72, 50.04)	
Age ≥50 years	111 (26.49%)	24 (32.00%)	87 (25.29%)	0.23	54 (28.13%)	57 (25.11%)	0.49
Ethnicity				0.053			**0.011**
Caucasian	339 (80.91%)	69 (92%)	270 (78.49%)		161 (83.85%)	178 (78.41%)	
Latin/Hispanic	19 (4.53%)	3 (4%)	16 (4.65%)		10 (5.21%)	9 (3.96%)	
Afro-american/Afro	34 (8.11%)	1 (1.33%)	33 (9.59%)		17 (8.85%)	17 (7.49%)	
Other	27 (6.44%)	2 (2.67%)	25 (7.26%)		4 (2.08%)	23 (10.13%)	
Caucasian ethnicity	339 (80.91%)	69 (92%)	270 (78.49%)	**0.007**	161 (83.85%)	178 (78.41%)	0.16
At least 1 coinfection	232 (55.37%)	41 (54.67%)	191 (55.52%)	0.89	109 (56.77%)	123 (54.19%)	0.60
HCV	34 (8.11%)	5 (6.67%)	29 (8.43%)	0.61	19 (9.90%)	15 (6.61%)	0.22
HBV	29 (6.92%)	0 (0%)	29 (8.43%)	**0.009**	8 (4.17%)	21 (9.25%)	0.041
HAV	13 (3.10%)	2 (2.67%)	11 (3.20%)	>0.99	7 (3.65%)	6 (2.64%)	0.56
EBV	36 (8.59%)	10 (13.33%)	26 (7.56%)	0.11	15 (7.81%)	21 (9.25%)	0.60
LUE	74 (17.66%)	21 (28%)	53 (15.41%)	**0.010**	35 (18.23%)	39 (17.18%)	0.78
CMV	57 (13.60%)	11 (14.67%)	46 (13.37%)	0.77	25 (13.02%)	32 (14.10%)	0.75
Number of comorbidities				0.82			0.54
0	197 (47.02%)	36 (48%)	161 (46.80%)		86 (44.79%)	111 (48.90%)	
1	140 (33.41%)	26 (34.67%)	114 (33.14%)		65 (33.85%)	75 (33.04%)	
2	53 (12.65%)	11 (14.67%)	42 (12.21%)		28 (14.58%)	25 (11.01%)	
3	19 (4.53%)	2 (2.67%)	17 (4.94%)		10 (5.21%)	9 (3.96%)	
4	8 (1.91%)	0 (0%)	8 (2.33%)		2 (1.04%)	6 (2.64%)	
5	1 (0.24%)	0 (0%)	1 (0.29%)		1 (0.52%)	0 (0%)	
6	1 (0.24%)	0 (0%)	1 (0.29%)		0 (0%)	1 (0.44%)	
At least 1 comorbidity	223 (53.22%)	39 (52%)	184 (53.49%)	0.81	107 (55.73%)	116 (51.10%)	0.34
At least 2 comorbidities	82 (19.57%)	13 (17.85%)	69 (20.34%)	0.59	41 (21.35%)	41 (18.06%)	0.40
Cardiovascular disease	65 (15.51%)	8 (10.67%)	57 (16.57%)	0.20	30 (15.63%)	35 (15.42%)	0.95
Concomitant pharmacological treatment	224 (53.46%)	33 (44%)	191 (55.52%)	0.070	96 (50%)	128 (56.39%)	0.19
CD4 count (copies/mm^3^) at diagnosis				0.093			**<0.01**
Median (Q1, Q3)	371 (160, 553)	427 (210, 569)	363 (159, 546)		308 (77, 496)	414 (235, 599)	
(Missing)	2	1	1		1	1	
CD4/CD8 ratio at diagnosis				0.27			**0.032**
Median (Q1, Q3)	0.44 (0.20, 0.82)	0.45 (0.26, 0.95)	0.42 (0.20, 0.79)		0.38 (0.19, 0.74)	0.47 (0.26, 0.90)	
(Missing)	79	10	69		37	42	
CD4 count (copies/mm^3^) at switch				**0.014**			0.01
Median (Q1, Q3)	569 (350, 818)	649 (470, 889)	551.50 (335.50, 805)		507.50 (274, 765.50)	603 (401, 892)	
CD4/CD8 ratio at switch				0.11			**0.017**
Median (Q1, Q3)	0.71 (0.39, 1.24)	0.81 (0.52, 1.39)	0.69 (0.38, 1.20)		0.66 (0.36, 1.07)	0.79 (0.47, 1.33)	

^a^ n (%).

^b^ Wilcoxon rank sum test; Pearson’s Chi-squared test; Fisher’s exact test.

Overall, 344 (82.1%) were on TT, and 227 (54.2%) on an STR regimen. Comparisons between DT and TT groups revealed significant differences in terms of Caucasian ethnicity (DT vs. TT: 92.00% vs. 78.49%; p = 0.007) and the presence of HBV infection (DT vs. TT: 0.00% vs. 8.43%; p = 0.009). Differences between MTR and STR groups included ethnic distribution (p = 0.011), HBV prevalence (MTR vs. STR: 4.17% vs. 9.25%; p = 0.041), and obesity (MTR vs. STR: 4.69% vs. 1.32%; p = 0.04).

At diagnosis, the median CD4^+^ T cell count was 371 cells/mm^3^, with DT PWH having a slightly higher count (DT vs. TT: 427 vs. 363; p = 0.093). PWH on STR had significantly higher CD4^+^ T cell counts than MTR (MTR vs. STR: 308 vs. 414; p < 0.01). At switch, the median CD4^+^ T cell count was significantly higher in DT than TT (DT vs. TT: 649 vs. 551.50; p = 0.014) and in STR than MTR (MTR vs. STR: 507.50 vs. 603; p = 0.01). The CD4/CD8 ratio followed a similar trend, with PWH on STR showing significantly higher values than MTR (MTR vs. STR: 0.66 vs. 0.79; p = 0.017).

**FIGURE 1:**
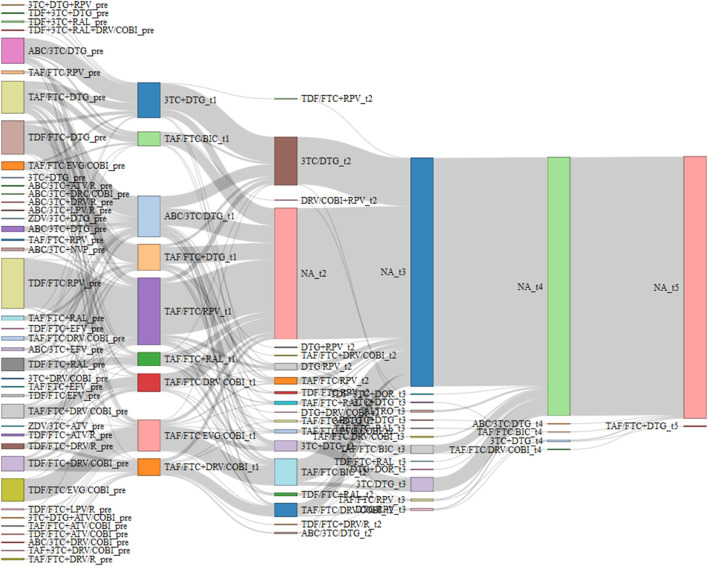
Sankey diagram of combination therapies that have at least 20 PWH at the first switch. Maximum 5 switch were recorded in follow up. NA bar represents PWH who have no longer switched.

The complexity of the broad landscape of combination therapies for HIV is depicted in the Sankey diagram (Figure 1). The Sankey diagram demonstrates the intricate nature of HIV treatment decision-making, with the 40 different therapeutic regimens at baseline, creating such a complex web of interconnections that discerning specific treatment trends becomes challenging. In the initial 24-month period, individuals with HIV averaged 1.41 treatment switches (SD 0.55). Selecting only therapies with at least 20 PWH at the first switch, there are nine different combinations. Among the PWHs included in the initial groups, those who switched a second time split into twice as many combinations (18).

In Table 2 the general characteristics of cART pre and post switch are described, including the pattern of switching. Before switching, 43.91% of people were on STR, with no significant differences between DT and TT (p = 0.12) but a significant difference between MTR and STR (p < 0.01). Nucleoside reverse transcriptase inhibitors (NRTIs) were the most frequently used drugs (98.57%), with TT PWH receiving them more commonly than DT (p < 0.01). DT PWH were more likely to use NSTIs (p = 0.022), and MTR PWH had higher NSTI usage than STR (p = 0.01). Optimization was the primary reason for switching (47.49%), with adherence improvement being a significant factor, especially in STR PWH (p < 0.01). Low-Dose Regimens (LDR) were significantly more frequent in DT than TT (DT vs. TT: 90.67% vs. 9.30%; p < 0.01) and in MTR than STR (MTR vs. STR: 35.94% vs. 13.66%; p < 0.01). Tenofovir Disoproxil Fumarate (TDF) to Tenofovir Alafenamide (TAF) switches were more common in TT than DT (DT vs. TT: 0% vs. 45.06%; p < 0.01). The median time from diagnosis to switch was 14.70 months, with less DT PWH switching within 6 months after diagnosis than TT (DT vs. TT: 13.33% vs. 25.29%; p = 0.026). Fixed-dose combination (FDCs) switches were more prevalence in TT than DT (DT vs. TT: 8.00% vs. 29.36%; p < 0.01).

**TABLE 2:** General pre and post switch characteristics.

		Comparison DT vs. TT			Comparison MTR vs. STR		
Characteristic	Overall N = 419^a^	DT N = 75^a^	TT N = 344^a^	p-value^b^	MTR N = 192^a^	STR N = 227^a^	p-value^b^
Pre-switch therapy DT/TT				**<0.01**			**0.026**
DT	8 (1.91%)	6 (8%)	2 (0.58%)		7 (3.65%)	1 (0.44%)	
TT	411 (98.09%)	69 (92%)	342 (99.42%)		185 (96.35%)	226 (99.56%)	
Pre-switch therapy MTR/STR				0.12			**<0.01**
MTR	235 (56.09%)	36 (48%)	199 (57.85%)		137 (71.35%)	98 (43.17%)	
STR	184 (43.91%)	39 (52%)	145 (42.15%)		55 (28.65%)	129 (56.83%)	
Pre-switch therapy – Anchor drug				**<0.01**			**<0.01**
INSTI	223 (53.24%)	64 (84.00%)	160 (46.52%)		115 (59.38%)	108 (47.14%)	
IP	107 (25.54%)	10 (13.33%)	97 (28.20%)		70 (36.46%)	37 (16.30%)	
NNRTI	89 (21.24%)	2 (2.67%)	87 (25.29%)		7 (3.65%)	82 (36.12%)	
Switch pattern				0.62			0.58
Intollerance	48 (11.46%)	9 (12%)	39 (11.34%)		27 (14.06%)	21 (9.25%)	
Risk of exacerbate present comorbidities	10 (2.39%)	2 (2.67%)	8 (2.33%)		3 (1.56%)	7 (3.08%)	
Prevention of long-term toxicity	199 (47.49%)	41 (54.67%)	158 (45.93%)		104 (54.17%)	95 (41.85%)	
Current regime no longer recommended	21 (5.01%)	2 (2.67%)	19 (5.52%)		8 (4.17%)	13 (5.73%)	
Drug interactions	10 (2.39%)	0 (0%)	10 (2.91%)		5 (2.60%)	5 (2.20%)	
Improve adherence	103 (24.58%)	15 (20%)	88 (25.58%)		25 (13.02%)	78 (34.36%)	
Better cost/effectiveness	23 (5.49%)	5 (6.67%)	18 (5.23%)		17 (8.85%)	6 (2.64%)	
Others	5 (1.19%)	1 (1.33%)	4 (1.16%)		3 (1.56%)	2 (0.88%)	
Clustered switch pattern: active and reactive				0.53			**<0.01**
Active	381 (90.93%)	69 (92.00%)	322 (93.60%)		172 (89.58%)	219 (96.48%)	
Reactive	28 (9.07%)	6 (8.00%)	22 (6.40%)		20 (10.42%)	8 (3.52%)	
Type of optimization				**<0.01**			**<0.01**
LDR	100 (23.87%)	68 (90.67%)	32 (9.30%)		69 (35.94%)	31 (13.66%)	
FDCs	107 (25.54%)	6 (8%)	101 (29.36%)		31 (16.15%)	76 (33.48%)	
TDF - TAF	155 (36.99%)	0 (0%)	155 (45.06%)		59 (30.73%)	96 (42.29%)	
Others	57 (13.60%)	1 (1.33%)	56 (16.28%)		33 (17.19%)	24 (10.57%)	
Time between diagnosis and switch (months)				0.36			0.79
Median (Q1, Q3)	14.70 (6.83, 34.33)	16.73 (9.63, 27.47)	13.97 (5.90, 37.60)		15.23 (7.33, 28.72)	14.47 (5.80, 36.27)	
Switch within 6 months after diagnosis	97 (23.15%)	10 (13.33%)	87 (25.29%)	**0.026**	40 (20.83%)	57 (25.11%)	0.30
Switch within 12 months after diagnosis	178 (42.48%)	26 (34.67%)	152 (44.19%)	0.13	80 (41.67%)	98 (43.17%)	0.76
Switch therapy DT/TT				**-**			**<0.01**
DT	75 (17.90%)				63 (32.81%)	12 (5.29%)	
TT	344 (82.10%)				129 (67.19%)	215 (94.71%)	
Switch therapy MTR/STR				**<0.01**			**-**
MTR	192 (45.82%)	63 (84.00%)	129 (37.50%)				
STR	227 (54.18%)	12 (16.00%)	215 (62.50%)				
Switch therapy – Anchor drug				**<0.01**			**<0.01**
INSTI	246 (58.71%)	72 (96.00%)	174 (50.58%)		129 (67.19%)	117 (51.54%)	
IP	76 (18.14%)	2 (2.67%)	74 (21.51%)		56 (29.17%)	20 (8.81%)	
NNRTI	97 (23.15%)	1 (1.33%)	96 (27.91%)		7 (3.65%)	90 (39.65%)	

^a^ n (%).

^b^ Wilcoxon rank sum test; Pearson’s Chi-squared test; Fisher’s exact test.

**FIGURE 2:**
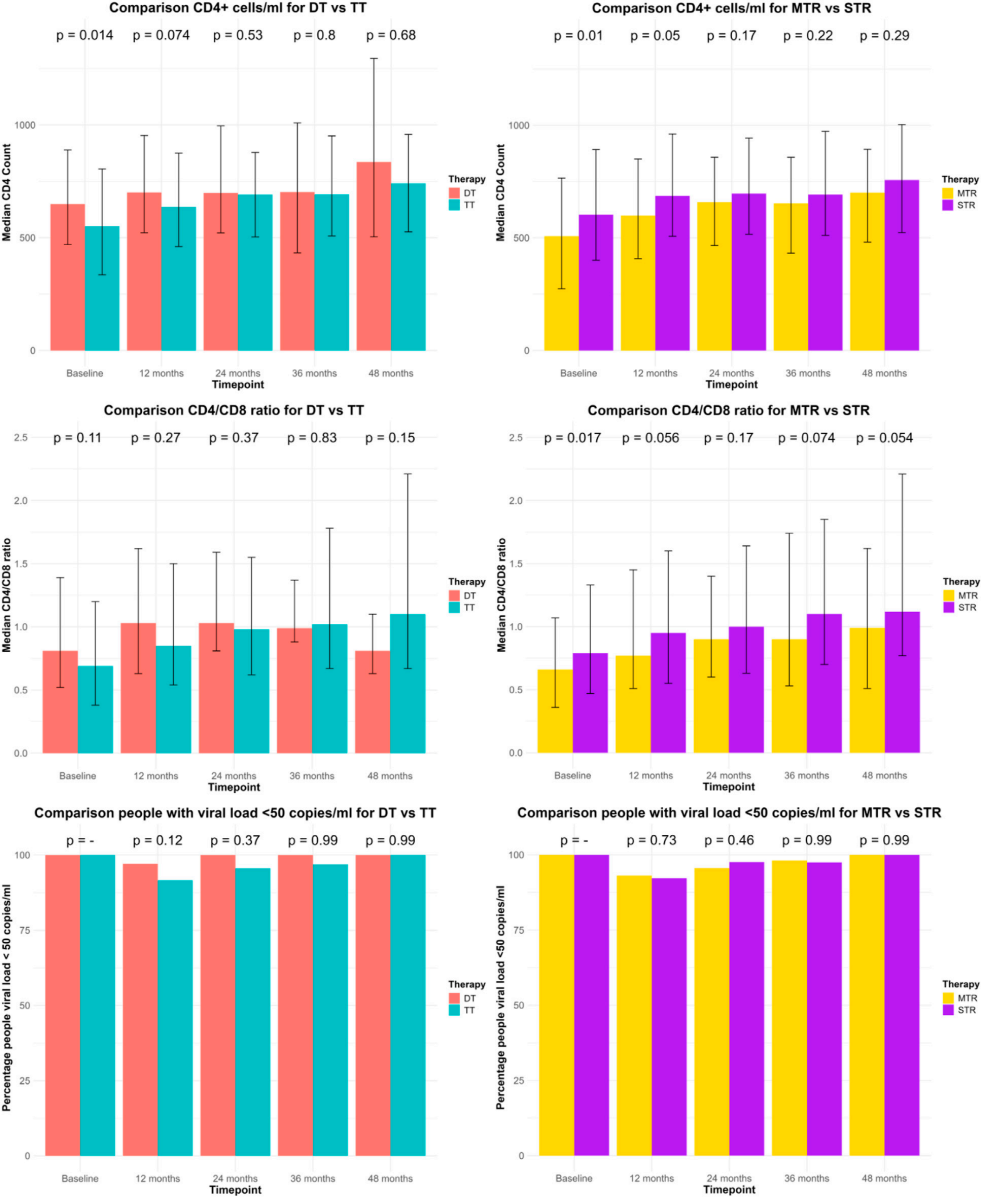
Description of the comparisons between DT vs. TT and MTR vs. STR through efficacy endpoints registered at baseline, 12-, 24-, 36- and 48-month post-switch. CD4^+^ counts cells/mL and CD4/CD8 ratio are represented through median and IQR. Number of persons living with HIV with viral load <50 copies/mL are represented through percentage.

The Supplementary Tables S1A–D describe the comparisons of DT vs. TT and MTR vs. STR through efficacy endpoints registered 12-, 24-, 36- and 48-month post-switch. 12 months post-switch. STR PWH had significantly higher CD4 counts than MTR (MTR vs. STR: 599.50 vs. 686.50; p = 0.05). Virological suppression was comparable across groups (DT: 97.1%, TT: 91.6%, p = 0.12; MTR: 93.1%, STR: 92.2%, p = 0.73). At 24 months, no significant differences were observed in CD4 counts, CD4/CD8 ratios, or virological suppression. However, MTR PWH showed a trend toward higher CD4 increases (MTR vs. STR: 92 vs. 50; p = 0.073). At 36 months, TT PWH exhibited greater CD4 increases than DT (DT vs. TT: 18.00 vs. 138.50; p = 0.042). At 48 months, MTR PWH had a significantly greater CD4 increase than STR (MTR vs. STR: 178.00 vs. 96.50; p = 0.017), while virological suppression remains high across all groups. These data are described in Figure 2.

The discontinuation data are described in Supplementary Table S2. Treatment discontinuation was primarily driven by optimization, with economic and tolerability concerns also playing a role. The median time to discontinuation was longer in TT than DT (DT vs. TT: 23.90 vs. 35.23; p < 0.01) and in STR than MTR (MTR vs. STR: 23.68 vs. 39.93; p < 0.01).

### 3.1 Propensity score matching analysis

Supplementary Figure S3A shows the balance plot of DT vs. TT with IPW Propensity score. Before adjustment, age ≥50, CD4 count ≥500, CD4/CD8 ratio ≥1 and Caucasian ethnicity exhibited significant differences between the two groups. After weighting, all covariates were effectively balanced, with SMDs reduced below the conventional threshold of 0.1, indicating a well-matched sample between the two treatment arms. Supplementary Table S3 shows an acceptable range of weights (DT: 1–1; TT: 0.048–0.458), a coefficient of variance acceptable (TT: 0.513) and a level of entropy low (0.138). As the IPW weights showed no extreme values and low variability, no further sensitivity analyses were deemed necessary to assess the robustness of the propensity score model.

Similarly, Supplementary Figure S3b describes the balance plot of MTR vs. STR. In this case, CD4 counts ≥500, CD4/CD8 ratio ≥1 and Caucasian ethnicity exceeding an SMD of 0.1. Propensity scores successfully mitigated these differences, achieving near-perfect balance across all covariates, with all SMDs approaching zero. Supplementary Table S3 shows an acceptable range of weights (MTR: 0.756–2.294; STR: 1–1), a coefficient of variance acceptable (STR: 0.284) and a level of entropy low (0.039). As the IPW weights showed no extreme values and low variability, no further sensitivity analyses were deemed necessary to assess the robustness of the propensity score model.

All details of Propensity scores are described in Supplementary Table S3.

### 3.2 Long-term immunovirological outcomes: multivariate analysis of ART regimens

Supplementary Table S4 presents the findings from weighted multivariate logistic regression models that were carefully adjusted through propensity score weighting to balance potential confounders and examine how treatment strategy affects key clinical outcomes over 48 months of follow-up.

When examining virological suppression rates, the comparison between DT and TT revealed interesting patterns. At the 12-month mark, both treatment approaches demonstrated comparable effectiveness in achieving viral suppression below 50 copies/mL, with no statistically significant difference observed (OR = 0.31, 95% CI: 0.05–2.11, p = 0.232). Remarkably, by 24 months and continuing through 36 and 48 months, all persons living with HIV receiving DT had achieved and maintained viral loads below 50 copies/mL, creating a scenario where statistical modeling could not converge due to the universal success in this group. The comparison between MTR and STR showed consistent virological outcomes across all time points, with no significant differences emerging throughout the study period.

The story of immune recovery, as measured by CD4 count restoration, followed a similar trajectory. Both DT and TT approaches demonstrated equivalent capacity for CD4 count recovery, with persons living with HIV in both groups showing comparable improvements in achieving normal CD4 counts (≥500 copies/mm^3^) throughout the 48-month observation period. This pattern of equivalence was mirrored in the MTR *versus* STR comparison, where tablet formulation appeared to have no meaningful impact on immune reconstitution. The CD4/CD8 ratio, an important marker of immune system normalization, demonstrated the same encouraging trend observed with CD4 count recovery, suggesting that treatment strategy did not significantly influence the restoration of immune balance in either comparison.

### 3.3Treatment persistence over time: Kaplan-Meier analysis of regimen durability

The weighted Kaplan-Meier analysis was performed for each comparison, considering time (in months) from switch to discontinuation due to reactive discontinuation (failure, toxicity, low adherence, drug interactions, drug resistance, or improved compliance).

**FIGURE 3:**
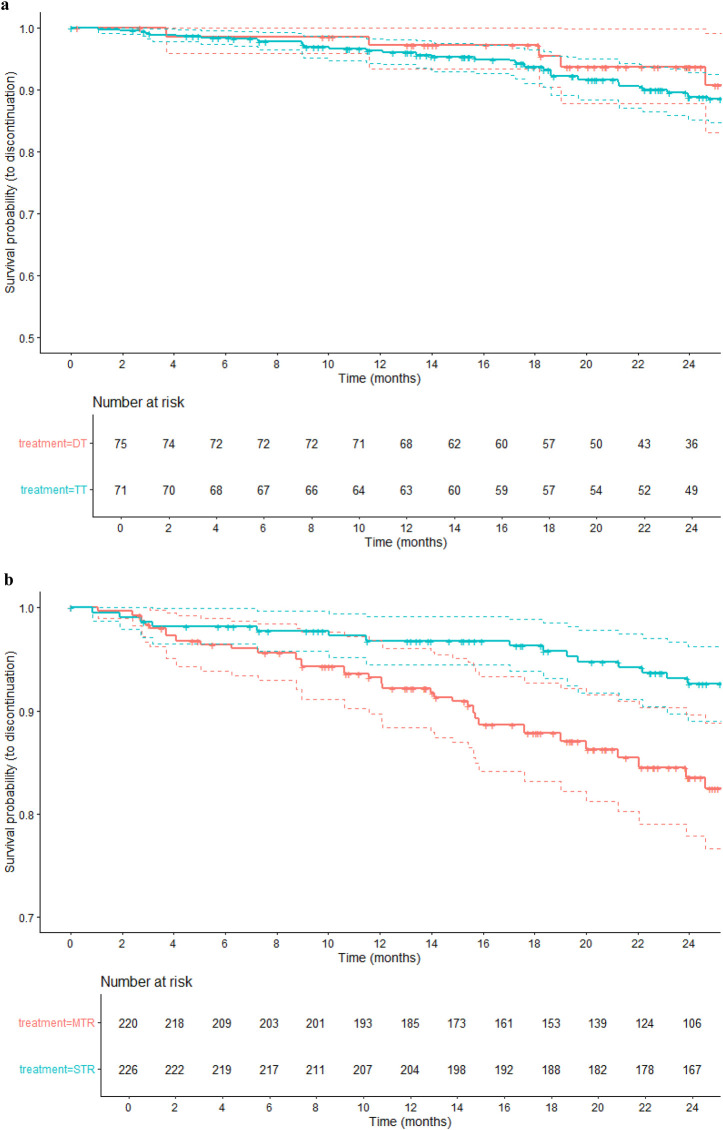
**(a)** Kaplan–Meier curves for time to treatment discontinuation: DT vs. TT (24-month follow-up). **(b)** Kaplan–Meier curves for time to treatment discontinuation: MTR vs. STR (24-month follow-up).

Figure 3a shows Kaplan Meier curves of comparison between DT vs. TT. At 24 months, the estimated probability of treatment persistence was 94% (88%–100%) in the DT group and 89% (85%–93%) in the TT group. The survival curves suggest a trend towards higher persistence in the DT group, though confidence intervals overlap at all time points.

Figure 3b shows Kaplan Meier curves of comparison between MTR vs. STR. At 24 months, the persistence probability was 93% (89%–96%) in STR group compared to 84% (78%–90%) in the MTR group. The STR group consistently showed higher treatment persistence across all follow-up periods.

To account for INSTI-based therapy, which was not captured in our initial propensity score, we performed weighted multivariate Cox regression analysis. Table 3 presents these survival analysis results, examining treatment durability across different therapeutic approaches.

**TABLE 3:** Results of multivariate Cox regression weighted by propensity score based on comparison DT vs. TT and MRT vs. STR, adjusted for INSTI-based therapy.

Characteristic	aHR^a^	95% CI^a^	p-value
TT (vs. DT)	1.33	0.59, 3.01	0.5
STR (vs. MTR)	0.56	0.32, 0.97	**0.039**

^a^ aHR, adjusted Hazard Ratio; CI, confidence interval.

The comparison between DT and TT showed no significant difference in discontinuation risk, with TT persons living with HIV having a slightly higher but non-significant aHR of 1.33 (95% CI: 0.59–3.01, p = 0.5). This suggests that the number of drugs in a regimen may not substantially impact treatment persistence.

In contrast, tablet formulation showed a meaningful effect on treatment durability. STR demonstrated significantly better persistence compared to MTR, with a 44% lower risk of treatment discontinuation (aHR = 0.56, 95% CI: 0.32–0.97, p = 0.039). This finding highlights the importance of formulation convenience in maintaining long-term treatment adherence among people living with HIV.

## 4 Discussion

Our work hypothesized that STRs and DT would maintain virological suppression comparable to standard regimens, but potentially demonstrate superior treatment persistence, especially for STRs’ options, in real-world settings. We observed similar virological and immunological outcomes across DT and TT groups, as well as between STR and MTR. However, STRs were significantly associated with greater treatment durability and lower rates of discontinuation. These results reinforce the potential of regimen optimization strategies to maintain clinical effectiveness while improving long-term persistence.

Moreover, this real-world study provides valuable insights into the effectiveness and durability of different ART strategies in PWH with viral load <50 copies/mL who underwent therapeutic optimization. Our findings contribute to the ongoing discourse on treatment simplification approaches by comparing both the number of antiretroviral agents (DT vs. TT) and the administration formulation (MTR vs. STR) in clinical practice across multiple Italian centers.

Our analysis of virological efficacy demonstrated that both DT and TT maintained high rates of virological suppression throughout the 48-month follow-up period, in agreement with those in the literature (Llibre et al., 2023; Russo et al., 2024; van Wyk et al., 2020a). While no significant difference was observed at 12 months, it is noteworthy that from 24 months onward, all persons living with HIV remaining on their initial switching therapy in both the DT and TT groups achieved complete virological suppression, resulting in non-convergence of the regression model. This suggests that, when appropriately selected, both therapeutic strategies can ensure durable virological control in real-world settings. Importantly, no significant differences in virological suppression were observed between MTR and STR approaches at any timepoint, suggesting that pill burden may have less impact on virological outcomes than previously thought, provided that persons living with HIV remain adherent to their prescribed regimen.

The durability analysis provided compelling evidence regarding treatment persistence. Although crude discontinuation rates differed between treatment groups (as shown in Supplementary Table S2), these findings must be interpreted with caution, as they do not account for time-to-event dynamics or censoring. In contrast, Kaplan-Meier and Cox regression analyses incorporate follow-up time and covariate adjustment, which may explain the lack of statistically significant differences observed in survival models. Therefore, in Cox models DT and TT showed comparable discontinuation rates, STR demonstrated significantly greater durability than MTR. This finding corroborates previous literature on the benefits of treatment simplification for improving persistence. Clay et al. (Clay et al., 2018) documented improved adherence with STRs, and our results extend this observation to demonstrate superior long-term durability in real-world clinical practice. The higher persistence observed in STR regimens may be partly explained by adherence-related discontinuations in the MTR group. In several cases, poor adherence led to a switch from MTR to STR, which is known to improve adherence due to reduced pill burden. This dynamic likely contributed to the more favorable persistence profile of STR regimens. The lack of significant difference in durability between DT and TT contrasts with concerns about the long-term sustainability of dual therapies and suggests that appropriately selected dual regimens can achieve comparable persistence to triple therapies.

The baseline characteristics of our study population revealed important differences that may influence treatment outcomes. PWH receiving DT had significantly higher CD4 counts at switch compared to those on TT, and similar advantages were observed in the STR *versus* MTR comparison. These baseline immunological differences reflect the careful patient selection practiced in clinical settings, where physicians may preferentially prescribe DT or STR to individuals with more favorable immunological profiles. Our use of propensity score weighting successfully balanced these baseline differences, enabling more reliable treatment comparisons.

The patterns of ART switching in our cohort provide valuable insights into clinical decision-making. Optimization (47.49%) and adherence improvement (24.58%) were the predominant reasons for regimen changes, highlighting the ongoing emphasis on treatment simplification in current HIV management. The significantly higher proportion of INSTI-based therapies in DT compared to TT reflects the growing reliance on integrase inhibitors as backbone agents in simplified regimens, consistent with current treatment guidelines (NIH, 2024; Saag et al., 2020).

Our study has several strengths that distinguish it from previous investigations. The multicenter design encompassing 20 centers enhances the generalizability of our findings. The extended follow-up period of 48 months provides valuable insights into the long-term outcomes of different treatment strategies, addressing a critical gap in the existing literature that often focuses on shorter durations. Additionally, our thorough statistical approach utilizing propensity score weighting minimized the impact of confounding factors that commonly affect observational studies. However, in the DT vs. TT comparison, the limited overlap in propensity score distributions - despite no extreme weights - suggests that the positivity assumption was only marginally satisfied. This represents a potential limitation affecting the robustness of the causal inference in this subgroup.

Nevertheless, certain limitations warrant consideration. The retrospective design introduces several potential biases, as treatment decisions were made by clinicians based on individual patient characteristics rather than randomization. Moreover, the requirement for at least 24 months of follow-up may have led to the inclusion of individuals with more stable treatment trajectories, potentially limiting the generalizability of the findings to those with earlier discontinuation or shorter follow-up durations. As the study was not population-based and relied on data from a network of selected centers, we cannot exclude the possibility that some persons living with HIV classified as lost to follow-up may have continued ART at non-participating facilities. This may have led to a degree of misclassification of censored observations and, potentially, a modest underestimation of treatment persistence. This observational approach may also be subject to information bias due to incomplete or inconsistent documentation in medical records, and temporal bias since treatment practices and available medications may have evolved during the study period. Additionally, unmeasured confounders such as socioeconomic factors, health literacy, social support systems, insurance coverage, and geographic accessibility to healthcare services could significantly influence treatment adherence and switching patterns, but were not captured in our analysis. Despite our efforts to control for confounding through propensity score weighting, residual confounding from these unmeasured variables cannot be completely eliminated. The positivity assumption may be limited, particularly in the DT *versus* TT comparison, where baseline covariate overlap between groups was suboptimal. This could affect the stability of weighted estimates and limits the strength of causal interpretation. While IPTW adjustment was applied, we caution against overinterpretation of effect estimates in this subgroup. Furthermore, our analysis did not include detailed pharmacokinetic data, comprehensive assessment of patient-reported quality of life outcomes, or measures of treatment satisfaction, which could provide additional context for the observed differences in treatment durability and help explain the underlying mechanisms driving treatment switches.

Furthermore, it is important to highlight that the landscape of antiretroviral therapies continues to evolve, with new drugs and combinations approved after our study period (2017–2019). In particular, combinations containing doravirine (DOR), a more recently approved NNRTI, represent an important area requiring further research (Mazzitelli et al., 2025; Orkin et al., 2021). Considering these DOR-based regimens, as well as the need for prospective studies are mandatory to validate these findings in broader populations and to assess the long-term effectiveness and durability of newer regimens.

In conclusion, our real-world study demonstrates that both DT and TT approaches can achieve and maintain high rates of virological suppression in appropriately selected people. The complete virological suppression observed in both groups from 24 months onward highlights the efficacy of both therapeutic strategies when properly implemented. STR showed significantly better durability than MTR, supporting the continued emphasis on treatment simplification. These findings contribute valuable evidence to guide clinicians in optimizing ART strategies for PWH with viral load <50 copies/mL, emphasizing the importance of individualized treatment approaches that consider both efficacy and patient-centered outcomes such as regimen complexity and tolerability. The complexity and diversity of potential treatment pathways, as vividly illustrated in the Sankey Diagram (Figure 1), underscores the critical importance of tailoring HIV pharmacological therapy to individual patient needs. This visualization captures the myriad combinations of regimens and switches implemented throughout the study, reinforcing that in the expansive landscape of HIV treatment options, a personalized approach remains paramount for optimizing long-term outcomes. Future research should focus on developing more STR options and evaluating their impact on long-term outcomes in diverse patient populations.

## Data Availability

The raw data supporting the conclusions of this article will be made available by the authors, without undue reservation.

## Group members of the SIFaCT MOSAICO Study Group

ASUGI - Azienda Sanitaria Universitaria Giuliano Isontina: Clara Palmieri, Stefano Loiacono, Andrea Misin; Azienda Ospedaliero-Universitaria di Cagliari: Arianna Cadeddu, Valentina Mureddu, Marila Camboni; Ospedale A. di Summa Perrino – Brindisi: Mariantonietta Piccoli, Anila Corja, Marina Allegrini; Azienda Ospedaliera Universitaria di Bologna - policlinico S. Orsola-Malpighi: Giulia Piazza, Salvatore Vitale, Cristina Bitello; Ospedale Centrale di Bolzano: Michela Falciani, Linda Cappellazzo, Raffaella Binazzi; Azienda Ospedaliera “SS. Antonio e Biagio e Cesare Arrigo” di Alessandria: Francesca Cammalleri, Giorgia Bo, Giuseppe Greco; Presidio Ospedaliero Santa Maria della Misericordia – AULSS Polesana, Rovigo: Roberta Rampazzo, Marina Malena; Ospedale Ss. Annunziata – Chieti: Nicola Petragnani; Presidio ospedaliero dell’Azienda AUSL Valle d’Aosta: Agostino Enza, Amadio Michele, Russo Ilaria; ASP Ragusa: Maria Rita Badagliacca, Salvatore Agosta, Dario Ventura; Azienda Ospedaliera Universitaria Integrata di Verona – Verona: Roberta Fraccaroli, Giovanna Gandini, Paola Marini; Ospedale San Bortolo – AULSS, Vicenza: Jenny Bolcato, Marta Cappellini, Giuliana Battagin; AUSL Piacenza: Sara Ferrari, Silvia Enisci, Aurora Scazzina; AOU Consorziale Policlinico di Bari - UO Farmacia: Francesco Pomarico, Maria Dell'Aera, Nicolò de Gennaro; Azienda Ospedaliera di Perugia: Martina Tegon, Veronica Gonzi, Anastasia Zoffoli, Sara Tordi; Ospedale “Infermi” di Rimini - AUSL Romagna: Lucia Rossi, Simonetta Palazzini, Kety Luzi; Ospedale Morgagni Pierantoni di Forlì - AUSL Romagna: Fabio Pieraccini, Marina Assirelli, Eleonora Magistrelli; Ospedale “Santa Maria delle Croci” di Ravenna – AUSL Romagna: Giovanna Rametta, Chiara Scolamacchia, Gabriele Fabbri; Azienda di Rilievo Nazionale ad Alta Specializzazione Garibaldi, Catania: Giuseppina Emanuela Fassari, Clara La Bruna, Claudia Ciraldo; AOUP Parma: Anna Gagliardi; University of Parma: Arianna Orcesi.
