# Supplementary material for: Durability and effectiveness of dual vs. triple therapy and tablet simplification in ART: findings from the Italian MOSAICO study
Source: Front Pharmacol. 2025 Aug 6;16:1633968. doi: 10.3389/fphar.2025.1633968 (PMC12365616; doi:10.3389/fphar.2025.1633968)
Supplement: Supplementary file 1 [file DataSheet1.docx]

Supplementary Material

**Table S1a**: Efficacy outcomes 12 months after the switch. Considered people who did not switch cART group until timepoints.

|  | | | Comparison DT vs TT | |  | Comparison MTR vs STR | |  |
| --- | --- | --- | --- | --- | --- | --- | --- | --- |
| **Characteristic** | | | **DT** N = 68*^1^* | **TT** N = 298*^1^* | **p-value***^2^* | **MTR** N = 160*^1^* | **STR** N = 204*^1^* | **p-value***^2^* |
| **CD4 count (cell/mm^3^)** | | |  |  | 0.074 |  |  | **0.05** |
| *Median (Q1, Q3)* | | | 701 (522.50, 953.50) | 636.50 (461, 875) |  | 599.50 (408, 850.50) | 686.50 (507, 961.50) |  |
| *(Missing)* | | | 4 | 0 |  | 4 | 0 |  |
| **CD4/CD8 ratio** | | |  |  | 0.27 |  |  | 0.056 |
| *Median (Q1, Q3)* | | | 1.03 (0.63, 1.62) | 0.85 (0.54, 1.50) |  | 0.77 (0.51, 1.45) | 0.95 (0.55, 1.60) |  |
| *(Missing)* | | | 5 | 11 |  | 7 | 9 |  |
| **HIV-RNA <50 copies/mL** | | | 66.0 / 68.0 (97.1%) | 273.0 / 298.0 (91.6%) | 0.12 | 149.0 / 160.0 (93.1%) | 188.0 / 204.0 (92.2%) | 0.73 |
| **Difference CD4 count (cell/mm^3^) from switch** | | |  |  | 0.37 |  |  | 0.39 |
| *Median (Q1, Q3)* | | | 31.50 (-52.50, 157.50) | 64 (-34, 158) |  | 56 (-66, 149) | 53.50 (-36, 168) |  |
| *(Missing)* | | | 4 | 0 |  | 4 | 0 |  |
| **Difference CD4/CD8 ratio from switch** | | |  |  | 0.75 |  |  | 0.29 |
| *Median (Q1, Q3)* | | | 0.10 (-0.01, 0.27) | 0.12 (0, 0.30) |  | 0.10 (0, 0.26) | 0.12 (0, 0.30) |  |
| *(Missing)* | | | 5 | 11 |  | 7 | 9 |  |
|  |  |  |  |  |  |  |  |  |
|  |  |  |  |  |  |  |  |  |

**Table S1b**: Efficacy outcomes 24 months after the switch. Considered people who did not switch cART group until timepoint.

|  | | | Terapia Primo Switch | | |  | Comparison MTR vs STR | |  |
| --- | --- | --- | --- | --- | --- | --- | --- | --- | --- |
| **Characteristic** | | | **dual** N = 36*^1^* | | **triplice** N = 228*^1^* | **p-value***^2^* | **MTR** N = 91*^1^* | **STR** N = 167*^1^* | **p-value***^2^* |
| **CD4 count (cell/mm^3^)** | | |  | |  | 0.53 |  |  | 0.17 |
| *Median (Q1, Q3)* | | | 698.50 (522, 996) | | 691.50 (503.50, 878) |  | 659 (466, 858) | 697 (516, 943) |  |
| *(Missing)* | | | 2 | | 0 |  | 2 | 0 |  |
| **CD4/CD8 ratio** | | |  | |  | 0.37 |  |  | 0.17 |
| *Median (Q1, Q3)* | | | 1.03 (0.81, 1.59) | | 0.98 (0.62, 1.55) |  | 0.90 (0.60, 1.40) | 1 (0.63, 1.64) |  |
| *(Missing)* | | | 6 | | 16 |  | 10 | 12 |  |
| **HIV-RNA <50 copies/mL** | | | 36.0 / 36.0 (100.0%) | | 218.0 / 228.0 (95.6%) | 0.37 | 87.0 / 91.0 (95.6%) | 163.0 / 167.0 (97.6%) | 0.46 |
| **Difference CD4 count (cell/mm^3^) from switch** | | |  | |  | 0.34 |  |  | 0.073 |
| *Median (Q1, Q3)* | | | 44.50 (-66, 134) | | 69.50 (-60, 196) |  | 92 (-19, 206) | 50 (-69, 179) |  |
| *(Missing)* | | | 2 | | 0 |  | 2 | 0 |  |
| **Difference CD4/CD8 ratio from switch** | | |  | |  | 0.51 |  |  | 0.58 |
| *Median (Q1, Q3)* | | | 0.20 (0.09, 0.41) | | 0.20 (0.01, 0.40) |  | 0.21 (0.05, 0.39) | 0.18 (0.01, 0.40) |  |
| *(Missing)* | | | 6 | | 16 |  | 10 | 12 |  |
|  |  |  | |  |  |  |  |  |  |
|  |  |  | |  |  |  |  |  |  |

**Table S1c**: Efficacy outcomes 36 months after the switch. Considered people who did not switch cART group until timepoint.

|  | | | Comparison DT vs TT | |  | Comparison MTR vs STR | |  |
| --- | --- | --- | --- | --- | --- | --- | --- | --- |
| **Characteristic** | | | **DT** N = 18*^1^* | **TT** N = 166*^1^* | **p-value***^2^* | **MTR** N = 56*^1^* | **STR** N = 124*^1^* | **p-value***^2^* |
| **CD4 count (cell/mm^3^)** | | |  |  | 0.80 |  |  | 0.22 |
| *Median (Q1, Q3)* | | | 702.50 (433, 1,009) | 692 (508, 951.50) |  | 653.50 (432, 858) | 692 (511, 973) |  |
| *(Missing)* | | | 4 | 6 |  | 6 | 4 |  |
| **CD4/CD8 ratio** | | |  |  | 0.83 |  |  | 0.074 |
| *Median (Q1, Q3)* | | | 0.99 (0.88, 1.37) | 1.02 (0.67, 1.78) |  | 0.90 (0.53, 1.74) | 1.10 (0.70, 1.85) |  |
| *(Missing)* | | | 4 | 13 |  | 8 | 10 |  |
| **HIV-RNA <50 copies/mL** | | | 18.0 / 18.0 (100.0%) | 154.0 / 159.0 (96.9%) | >0.99 | 53.0 / 54.0 (98.1%) | 116.0 / 119.0 (97.5%) | >0.99 |
| *(Missing)* | | | 0 | 7 |  | 2 | 5 |  |
| **Difference CD4 count (cell/mm^3^) from switch** | | |  |  | **0.042** |  |  | 0.052 |
| *Median (Q1, Q3)* | | | 18 (-30, 119) | 138.50 (-28, 252.50) |  | 178 (23, 276) | 105 (-48, 236) |  |
| *(Missing)* | | | 4 | 6 |  | 6 | 4 |  |
| **Difference CD4/CD8 ratio from switch** | | |  |  | 0.49 |  |  | 0.82 |
| *Median (Q1, Q3)* | | | 0.32 (0.11, 0.60) | 0.24 (0.07, 0.58) |  | 0.23 (0.08, 0.54) | 0.24 (0.05, 0.60) |  |
| *(Missing)* | | | 4 | 13 |  | 8 | 10 |  |
|  |  |  |  |  |  |  |  |  |
|  |  |  |  |  |  |  |  |  |

**Table S1d**: Efficacy outcomes 48 months after the switch. Considered patients who did not switch cART group until timepoint**.**

|  | | | Comaparison DT vs TT | |  |  | Comparison MTR vs STR |  |
| --- | --- | --- | --- | --- | --- | --- | --- | --- |
| **Characteristic** | | | **DT** N = 7*^1^* | **TT** N = 111*^1^* | **p-value***^2^* | **MTR** N = 37*^1^* | **STR** N = 79*^1^* | **p-value***^2^* |
| **CD4 count (cell/mm^3^)** | | |  |  | 0.68 |  |  | 0.29 |
| *Median (Q1, Q3)* | | | 836 (504, 1,295) | 741 (526, 958) |  | 701 (481.65, 893) | 757 (523.50, 1,003) |  |
| *(Missing)* | | | 0 | 6 |  | 4 | 3 |  |
| **CD4/CD8 ratio** | | |  |  | 0.15 |  |  | 0.054 |
| *Median (Q1, Q3)* | | | 0.81 (0.63, 1.10) | 1.10 (0.67, 2.21) |  | 0.99 (0.51, 1.62) | 1.12 (0.77, 21) |  |
| *(Missing)* | | | 0 | 7 |  | 4 | 4 |  |
| **HIV-RNA <50 copies/mL** | | | 7.0 / 7.0 (100.0%) | 104.0 / 104.0 (100.0%) |  | 32.0 / 32.0 (100.0%) | 76.0 / 76.0 (100.0%) | 0.99 |
| *(Missing)* | | | 0 | 7 |  | 5 | 3 |  |
| **Difference CD4 count (cell/mm^3^) from switch** | | |  |  | 0.76 |  |  | **0.017** |
| *Median (Q1, Q3)* | | | 60 (17, 216) | 137 (4, 248) |  | 178 (98, 359) | 96.50 (-14, 205.50) |  |
| *(Missing)* | | | 0 | 6 |  | 4 | 3 |  |
| **Difference CD4/CD8 ratio from switch** | | |  |  | 0.75 |  |  | 0.74 |
| *Median (Q1, Q3)* | | | 0.20 (0.13, 0.56) | 0.30 (0.10, 0.76) |  | 0.32 (0.17, 0.42) | 0.30 (0.10, 0.92) |  |
| *(Missing)* | | | 0 | 7 |  | 4 | 4 |  |
|  |  |  |  |  |  |  |  |  |
|  |  |  |  |  |  |  |  |  |

**Table S2:** Therapy discontinuation. Description of switch pattern and time to switch when patients switch to opposite switch group cART respect first switch cART

|  | Comparison DT vs TT |  | | Comparison MTR vs STR | |  | |
| --- | --- | --- | --- | --- | --- | --- | --- |
| **Characteristic** | **DT** N = 75*^1^* | **TT** N = 344*^1^* | **p-value***^2^* | **MTR** N = 192*^1^* | **STR** N = 227*^1^* | | **p-value***^2^* |
| **Discontinuation  pattern** |  |  | 0.11 |  |  | | 0.18 |
| *Virological failure* | 0 (0%) | 2 (1.13%) |  | 3 (2.27%) | 1 (1.04%) | |  |
| *Toxicity/Adverse event* | 6 (11.76%) | 29 (16.38%) |  | 13 (9.85%) | 18 (18.75%) | |  |
| *Poor adherence* | 0 (0%) | 4 (2.26%) |  | 3 (2.27%) | 1 (1.04%) | |  |
| *Drug interaction* | 1 (1.96%) | 10 (5.65%) |  | 2 (1.52%) | 5 (5.21%) | |  |
| *Drug resistance* | 0 (0%) | 2 (1.13%) |  | 0 (0%) | 0 (0%) | |  |
| *Cost-effectiveness* | 0 (0%) | 12 (6.78%) |  | 1 (0.76%) | 11 (11.46%) | |  |
| *Improve compliance* | 1 (1.96%) | 9 (5.08%) |  | 13 (9.85%) | 2 (2.08%) | |  |
| *Optimization* | 41 (80.39%) | 94 (53.11%) |  | 92 (69.70%) | 49 (51.04%) | |  |
| *Others* | 2 (3.92%) | 15 (8.47%) |  | 5 (3.79%) | 9 (9.38%) | |  |
| *No more switch* | 24 | 167 |  | 60 | 131 | |  |
| **Clustered discontinuation  pattern: active and reactive** |  |  | **<0.01** |  |  | | 0.20 |
| *Active* | 41 (80.39%) | 110 (59.89%) |  | 93 (70.46%) | 60 (52.50%) | |  |
| *Reactive* | 10 (19.61%) | 71 (41.11%) |  | 39 (29.54%) | 36 (47.50%) | |  |
| *No more switch* | 24 | 167 |  | 60 | 131 | |  |
| **Time to discontinuation** |  |  | **<0.01** |  |  | | **<0.01** |
| *Median (Q1, Q3)* | 23.90 (18.13, 35.23) | 35.23 (19.27, 53.40) |  | 23.67 (15.03, 42.07) | 39.93 (23.13, 53.27) | |  |
| *^1^*n (%) | | | | |  |  |  |
| *^2^*Pearson's Chi-squared test; Fisher's exact test; Wilcoxon rank sum test | | | | |  |  |  |

**Figure S1a**: Propensity score IPW DT vs TT


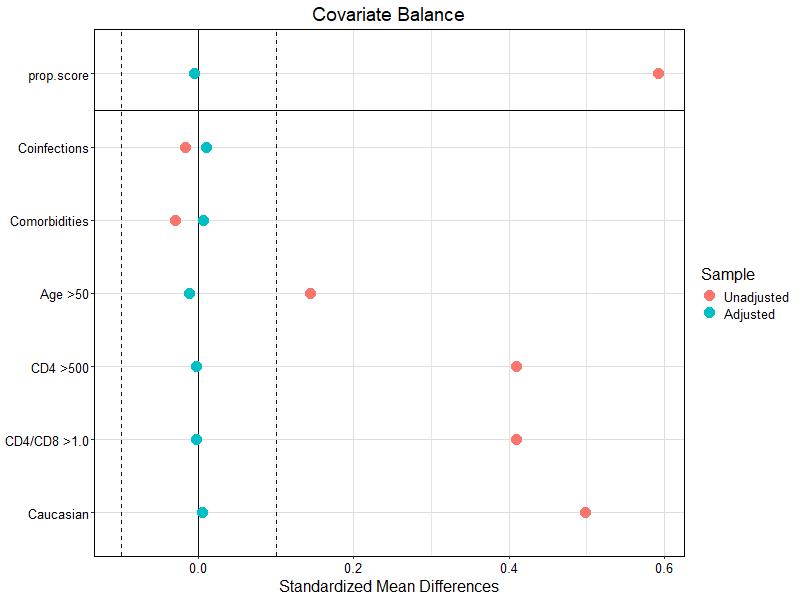


**Figure S1b**: Propensity score IPW MTR vs STR


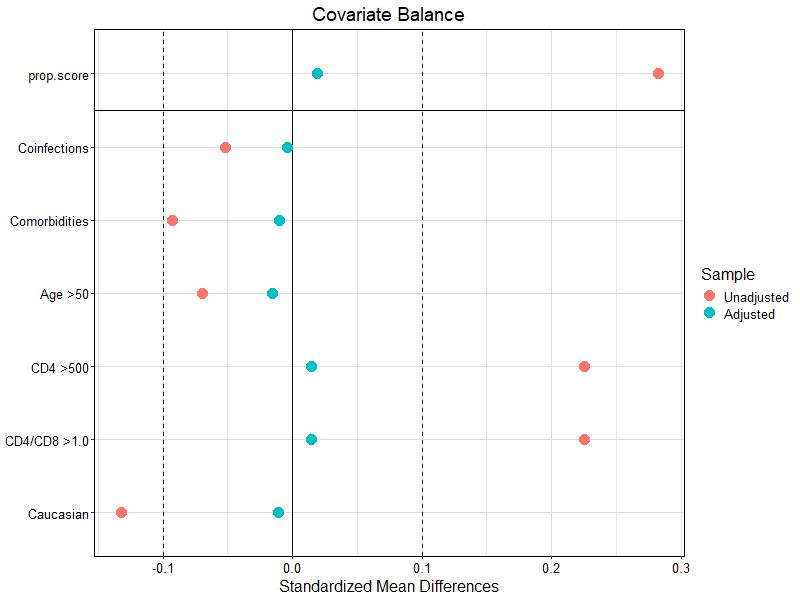


**Figure S2a:** Density plot IPW DT vs TT

**
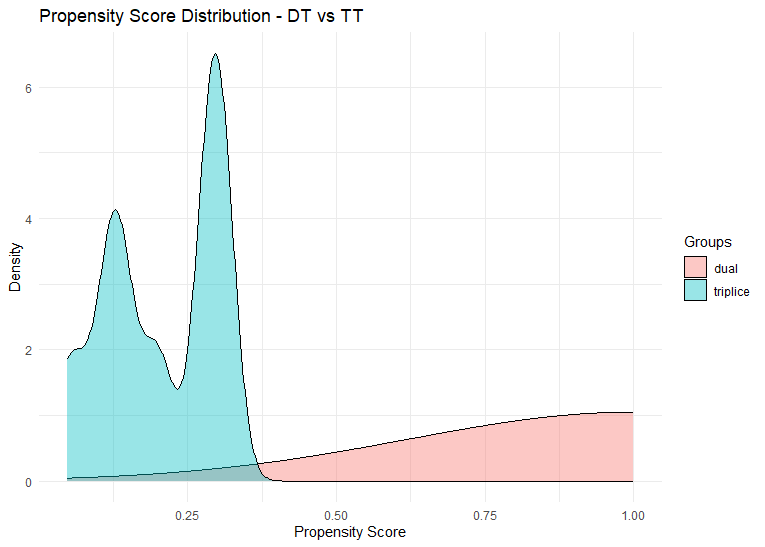
**

**Figure S2b:** Density plot IPW MTR (0) vs STR (1)

**
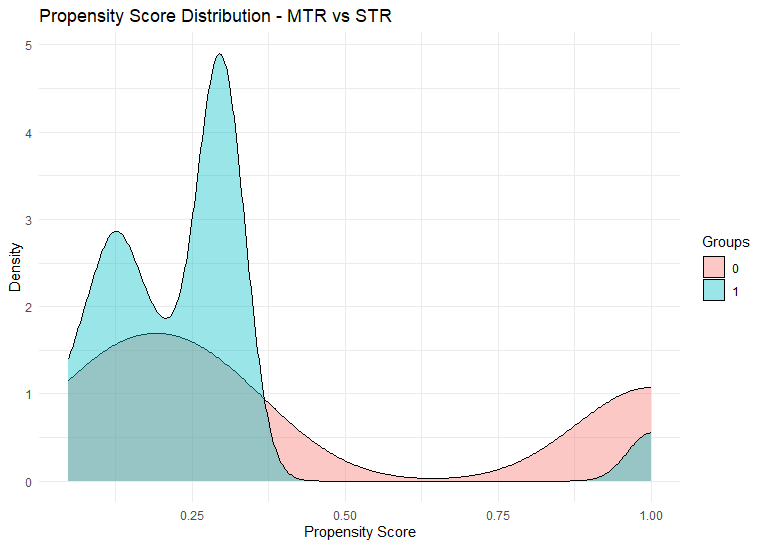
**

**Figure S3a:** Comparison of Propensity Score Distributions: Pre- and Post-Weighting. DT vs TT

**
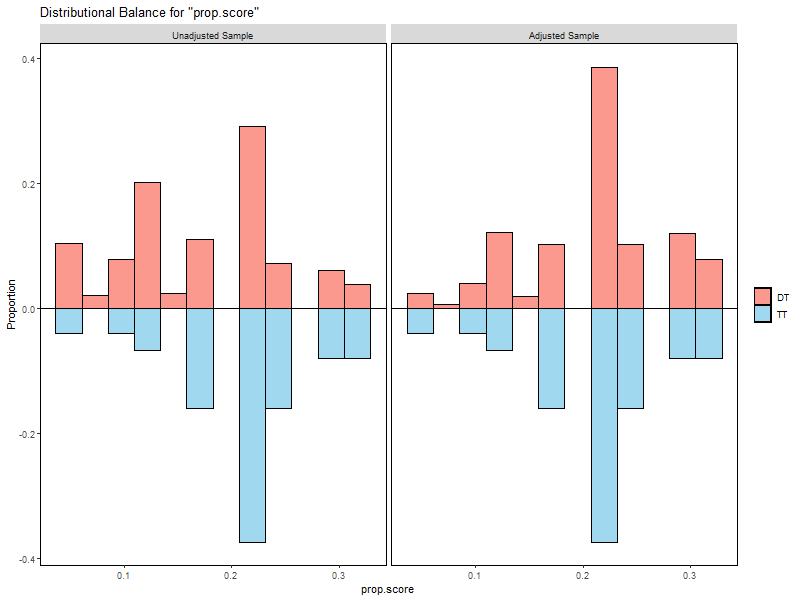
**

**Figure S3b:** Comparison of Propensity Score Distributions: Pre- and Post-Weighting. MTR vs STR

**
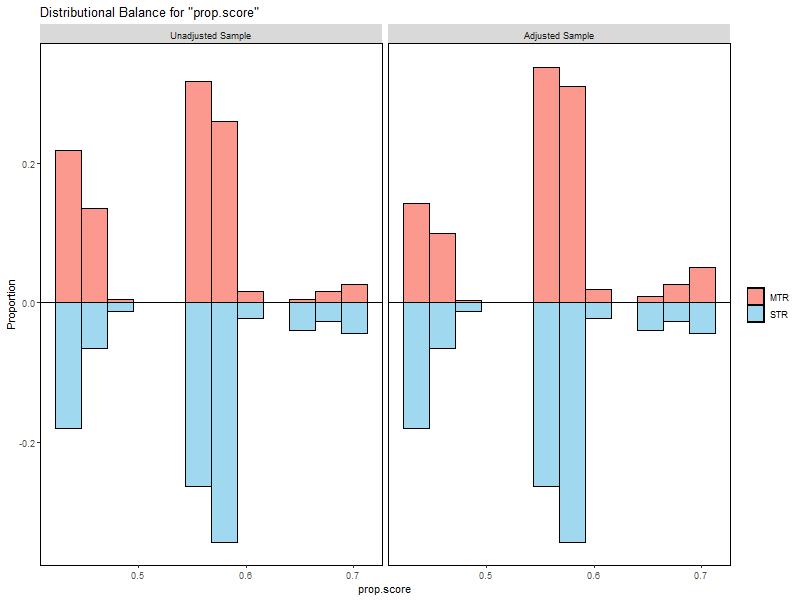
**

**Table S3**: Propensity score data DT vs TT and MTR vs STR

| **DT vs TT** | **DT** | **TT** |
| --- | --- | --- |
| Weight ranges (Min - Max) | 1 - 1 | 0.048 - 0.4577 |
| Coefficient of Variance | 0 | 0.513 |
| MAD (Mean Absolute Deviation) | 0 | 0.446 |
| Entropy | 0 | 0.138 |
| Zeros | 0 | 0 |
| ESS (Effective Sample Sizes) | 272.4 | 75 |
| **MTR vs STR** | **MTR** | **STR** |
| Weight ranges (Min - Max) | 0.7562 - 2.2936 | 1 - 1 |
| Coefficient of Variance | 0.284 | 0 |
| MAD (Mean Absolute Deviation) | 0.225 | 0 |
| Entropy | 0.039 | 0 |
| Zeros | 0 | 0 |
| ESS (Effective Sample Sizes) | 177.71 | 227 |

**Table S4**: Results of weighted multivariate logistic regression models. The outcomes are adjusted through propensity score weighting based on comparison DT vs TT and MTR vs STR to balance potential confounders, to investigate the impact of comparisons on people with viral load copies/mL < 50, normal CD4 count (CD4 count ≥500 copies/mm3), and normal CD4/CD8 ratio (CD4/CD8 ≥1) over a follow-up period of 48 months.

|  | **DT vs TT** | | | | **MTR vs STR** | | | |
| --- | --- | --- | --- | --- | --- | --- | --- | --- |
| **Virological suppression (HIV-RNA copies/mL <50)** | | | | | | | | |
| **Timepoint** | **Characteristic** | **OR***^1^* | **95% CI***^1^* | **p-value** | **Characteristic** | **OR***^1^* | **95% CI***^1^* | **p-value** |
| 12 months | TT (vs DT) | 0.31 | 0.05, 2.11 | 0.232 | STR (vs MTR) | 1.12 | 0.46, 2.71 | 0.800 |
|  | NSTI-based therapy | 0.84 | 0.36, 1.98 | 0.694 | NSTI-based therapy | 1.05 | 0.46, 2.40 | 0.903 |
| 24 months | TT (vs DT) | - | - | - | STR (vs MTR) | 3.47 | 0.71, 17.1 | 0.125 |
|  | NSTI-based therapy | 1.30 | 0.30, 5.62 | 0.727 | NSTI-based therapy | 2.07 | 0.40, 10.9 | 0.386 |
| 36 months | TT (vs DT) | - | - | - | STR (vs MTR) | 0.58 | 0.06, 5.94 | 0.648 |
|  | NSTI-based therapy | 0.44 | 0.03, 7.08 | 0.562 | NSTI-based therapy | 0.21 | 0.02, 2.05 | 0.176 |
| 48 months | TT (vs DT) | - | - | - | STR (vs MTR) | 1.07 | 0.67, 1.71 | 0.788 |
|  | NSTI-based therapy | 1.07 | 0.67, 1.70 | 0.767 | NSTI-based therapy | 1.02 | 0.66, 1.59 | 0.923 |
| **Normal CD4 count (CD4 count ≥500 copies/mm³)** | | | | | | | | |
| **Timepoint** | **Characteristic** | **OR***^1^* | **95% CI***^1^* | **p-value** | **Characteristic** | **OR***^1^* | **95% CI***^1^* | **p-value** |
| 12 months | TT (vs DT) | 0.69 | 0.29, 1.61 | 0.388 | STR (vs MTR) | 1.38 | 0.84, 2.27 | 0.205 |
|  | NSTI-based therapy | 0.66 | 0.30, 1.44 | 0.298 | NSTI-based therapy | 0.68 | 0.41, 1.14 | 0.141 |
| 24 months | TT (vs DT) | 1.23 | 0.41, 3.76 | 0.710 | STR (vs MTR) | 1.15 | 0.61, 2.16 | 0.670 |
|  | NSTI-based therapy | 1.22 | 0.42, 3.54 | 0.717 | NSTI-based therapy | 0.66 | 0.35, 1.26 | 0.204 |
| 36 months | TT (vs DT) | 2.45 | 0.62, 9.63 | 0.199 | STR (vs MTR) | 1.27 | 0.54, 2.98 | 0.580 |
|  | NSTI-based therapy | 1.74 | 0.55, 5.52 | 0.343 | NSTI-based therapy | 1.17 | 0.50, 2.73 | 0.710 |
| 48 months | TT (vs DT) | 0.58 | 0.05, 6.37 | 0.655 | STR (vs MTR) | 0.73 | 0.24, 2.24 | 0.579 |
|  | NSTI-based therapy | 0.58 | 0.19, 1.79 | 0.343 | NSTI-based therapy | 0.45 | 0.16, 1.29 | 0.137 |
| **Normal CD4/CD8 ratio (CD4/CD8 ≥1)** | | | | | | | | |
| **Timepoint** | **Characteristic** | **OR***^1^* | **95% CI***^1^* | **p-value** | **Characteristic** | **OR***^1^* | **95% CI***^1^* | **p-value** |
| 12 months | TT (vs DT) | 0.71 | 0.36, 1.39 | 0.312 | STR (vs MTR) | 1.41 | 0.87, 2.28 | 0.157 |
|  | NSTI-based therapy | 0.72 | 0.42, 1.21 | 0.208 | NSTI-based therapy | 0.68 | 0.42, 1.10 | 0.114 |
| 24 months | TT (vs DT) | 0.90 | 0.39, 2.10 | 0.806 | STR (vs MTR) | 1.33 | 0.73, 2.43 | 0.348 |
|  | NSTI-based therapy | 0.61 | 0.33, 1.12 | 0.109 | NSTI-based therapy | 1.05 | 0.60, 1.84 | 0.852 |
| 36 months | TT (vs DT) | 1.36 | 0.42, 4.36 | 0.606 | STR (vs MTR) | 1.78 | 0.82, 3.85 | 0.141 |
|  | NSTI-based therapy | 0.65 | 0.28, 1.50 | 0.313 | NSTI-based therapy | 0.92 | 0.45, 1.88 | 0.822 |
| 48 months | TT (vs DT) | 3.31 | 0.62, 17.8 | 0.161 | STR (vs MTR) | 1.19 | 0.47, 2.97 | 0.713 |
|  | NSTI-based therapy | 0.65 | 0.26, 1.66 | 0.369 | NSTI-based therapy | 0.58 | 0.24, 1.42 | 0.233 |

*^1^*OR = Odds Ratio, CI = Confidence Interval
